# Prevalence of metabolic syndrome and its risk factors among rural adults in Nantong, China

**DOI:** 10.1038/srep38089

**Published:** 2016-11-30

**Authors:** Jing Xiao, Chuan-Li Wu, Yue-Xia Gao, Shu-Lan Wang, Lei Wang, Qing-Yun Lu, Xiao-Jian Wang, Tian-Qi Hua, Huan Shen, Hui Cai

**Affiliations:** 1 Department of Epidemiology and Medical Statistics, School of Public Health, Nantong University, Nantong, Jiangsu, P.R. China; 2 Rugao Center for Diseases Prevention and Control, Nantong, Jiangsu, P.R. China; 3 Department of Chronic Disease and Prevention, Center for Disease Control and Prevention of Haian, Nantong, Jiangsu, P.R. China; 4 Division of Epidemiology, Vanderbilt University Medical Center, 2525 West End Avenue, 6th floor, Nashville, TN, 37203-1738, USA

## Abstract

The prevalence of metabolic syndrome (MS) varies worldwide due to genetic and environmental factors. A population-based cross-sectional study, with 37,582 participants recruited in Nantong, China in 2007-2008 (stage I) and 2013 (stage II). Socio-demographic, lifestyle factors, disease history and fasting blood sample were collected. The prevalence of MS was much higher in 2013 (42.6%) than that in 2007-2008 (21.6%), which was significantly higher in older people in both stages. Participants with two or more familial history of diseases were associated with a higher MS prevalence compared to those who didn’t have familial history of diseases. Total physical activity (PA) was associated with 24 and 19% decreased risk of MS in men and women. Occupational PA in moderate and vigorous intensity was associated with a 25%-45% decreased risk of MS. Female smokers who smoked more than 10 cigarettes per day or over 25 years were associated with 96 and 74% increased MS risk, respectively. The highest quartile of rice wine consumption was associated with a lower risk of MS with OR of 0.63 in women, compared with female non-drinkers. These findings indicated that PA and rice wine are two protective factors in MS prevention in rural areas of East China.

Metabolic syndrome (MS), defined as a cluster of metabolic risk factors including central obesity, dysglycemia, reduced high-density lipoprotein cholesterol (HDL-c), elevated triglycerides (TG) and hypertension^1^. It has increased two-fold the risk of cardiovascular disease and five- to nine-fold the risk of type 2 diabetes, compared with those who did not have MS^2^,^3^. In addition, MS has a 40% increased risk of cardiovascular disease mortality^4^,^5^, and the prevalence of MS has varied around 20–45% worldwide^6^,^7^. In China the prevalence has been reported to be 10.6% and 4.3% in urban and rural areas in 2002^8^. However, MS prevalence in rural China has experienced a dramatic increase, and reached 24.2% in 2014 because of rapid economic progress and lifestyle transitions in China^9^.

Lifestyles, behaviors and genetic factors may contribute to the prevalence of MS. Several previous studies found that physical activity (PA), cigarette smoking and alcohol consumption were associated with MS, but the conclusions are inconsistent^10^,^11^. Furthermore, some recent studies in China have reported the prevalence of MS and related risk factors, including smoking, alcohol consumption, lack of physical activity and familial history of disease, among urban people^12^,^13^ and rural people^2^,^14^, respectively. However, the results of these Chinese reports were inconsistent in different provinces and the prevalence varied geographically^15^.

In current study, a two-stage population-based cross-sectional study, 20,502 participants were recruited during 2007–2008 (stage I) and 17,080 participants were recruited in 2013 (stage II). A total of 37,582 participants aged 18–91 years were enrolled in the rural area of Nantong, China. The definition for MS has been proposed by the Joint Interim Statement (JIS) criteria in 2009^16^ using an ethnic-specific cut point for waist circumference as the central obesity criterion. The prevalence of MS among rural people in East China was estimated and the associations of smoking status, alcohol consumption, familial history of disease and PA with the prevalence of MS were evaluated. To the best of our knowledge, this is the largest MS study among people in rural China.

## Materials and Methods

### Participants

In our study, 43,515 residents aged 18–91 years from four townships in rural Nantong were invited to participate the study by trained interviewers through in-person interview. A total of 37,582 participants were enrolled in our study. Among them 20,502 participants (6,997 men and 13,505 women) were recruited from Liuqiao and Shigang townships between July 2007 and August 2008 with a response rate of 83.6%, and 17,080 participants (7,805 men and 9,275 women) were recruited from Haian and Rugao townships between March 2013 and November 2013 with the response rate of 89.9%. The details of the data in stage I have been described elsewhere^17^,^18^. The whole 37,582 participants with a response rate of 86.4%, the reasons for non-participation were refusal (2.9%), out of area during enrollment (7.2%), and other miscellaneous reasons including poor health or hearing problems (3.5%). A rural area was defined as an area with a primary administrative unit named a ‘village’. Most participants (99.5%) in our study lived in one of those villages at the time of interview and approximately 59.93% (8,871) men and 71.14% (16,205) women were farmers. The Boards of Scientific Research of Nantong University and the Nantong Centers for Disease Control ethics review board approved the ethical protocols and the informed consent was obtained for all participants in this study. And that all experimental protocols were carried out in accordance with approved guidelines.

### Data collection

Data of socio-demographic characteristics, lifestyle behaviors (including alcohol consumption, smoking status, PA at work and leisure time), personal medical history, and familial history of chronic diseases were collected using the standard questionnaires for all participants. Height, weight and waist circumference were measured and a blood sample was collected for biochemical measurements.

### PA

We used a questionnaire similar to the International Physical Activity Questionnaire, an instrument designed primarily for population surveillance activity^19^ with good validity and reliability^20^. We collected the cumulative hours per day spend on light-, moderate- and vigorous-intensity PA in occupational and leisure-time during the latest 7 days of the survey. Intensity levels of both leisure time PA (LPA) and occupational PA (OPA) were classified into four categories: LPA: no LPA (watching TV, reading and writing), light intensity LPA (Qi Gong and some stretching exercises), moderate intensity LPA (jogging and dancing) and vigorous intensity LPA (playing basketball, badminton); OPA: No job or sedentary job (typist, computer operator), light intensity OPA (clerk, teacher), moderate intensity OPA (driver, electrician) and vigorous intensity OPA (farmer, porter). Furthermore, we tertiled the hours per week of moderate- and vigorous-intensity PA in the analysis, respectively: vigorous intensity PA: no, low (≤19 hours/week (h/w) for men and ≤ 28 h/w for women) and high (>19 h/w for men and>28 h/w for women); moderate intensity PA: no, low (≤21 h/w for men and ≤ 17.5 h/w for women) and high (>21 h/w for men and >17.5 h/w for women). Total PA was calculated by sum of energy expenditure of three PA intensities and presented as metabolic equivalents-hours per week (MET-h/w). MET-h/w values were computed as hours of each PA intensity per week multiplied by its energy requirement: light intensity = 3.3 METs (based on walking METs), moderate intensity = 4.0 METs and vigorous intensity = 8.0 METs^19^. Total PA in MET-h/w was also classified into three categories based on the tertile categorization and definition as below: total PA: low (≤110.9 MET-h/w for men and ≤130.0 MET-h/w for women), medium (≤245.3 MET-h/w for men and ≤268.8 MET-h/w for women) and high (>245.3 MET-h/w for men and >268.8 MET-h/w for women).

### Smoking, alcohol consumption and socio-demographic factors

Smokers were defined as participants who had smoked at least 100 cigarettes in their lifetime. We collected age of participants who started and stopped smoking and how many cigarettes they consumed per day. Participants were classified into four categories based on quartiles of their smoking intensity by sex: non-smokers, ≤10, 20 and >20 cigarettes per day for men; non-smokers, ≤5, ≤10 and >10 cigarettes per day for women. Similar categorization was used for the years of smoke. Also we asked participants about their monthly alcohol consumption, including grape wine, rice wine, beer and liquor within a recent year. Among alcohol consumers in our study, only 1.1% of them consumed grape wine. Thus we only considered the beer, liquor and rice wine consumers in the analysis. One drink was defined as consume approximately 0.5 ounce of absolute alcohol, i.e. 4.8-ounce of rice wine, 12-ounce can of beer or 1 ounce of liquor^18^. Based on tertiles of alcohol intake of each type among drinkers, we classified the participants into 4 groups: non-drinkers, light, moderate and heavy drinkers.

### Anthropometric and biochemical measurements

All participants were collected anthropometric data and blood sample at in-person interviewed by well-trained interviewers using a standard protocol. Weight, height and waist circumference around the navel were measured twice for each participant to prevent reading and typing errors. A third measurement was taken if difference between two measurements was larger than 1 cm for height and waist circumference or 1 kilogram (kg) for weight. Then the average of two closely measurements in height, weight, and waist circumference was used in our study. Body mass index (BMI) was calculated using body weight in kilograms divided by the square of body height in meters.

A 10-ml blood sample for each participant, following an overnight fast, was drawn into an EDTA vacutainer tube at in-person interview, which was stored in a portable Styrofoam box with ice packs (0–4 °C) and transported to a laboratory of Nantong Centers for Disease Control or Nantong University within 8 hours. An automated chemistry analyzer (Hitachi 7180, Tokyo, Japan) used to measure glucose and lipid levels within 6-hour serum sample separated by centrifugation of the blood sample. Reagents from the Shino-Test Corporation in Japan were used to enzymatically analyze. The remaining samples were stored at −70 °C for additional laboratory assays. The variation coefficient of glucose, HDL-c and TG levels in both inter- and intra- assay were less than 3.5%.

Other socio-demographic factors, such as age at interview, education (none/elementary school, middle school, high school/college and above), marital status and occupation were treated as potential confounders in our study.

### Criteria of metabolic syndrome

MS was defined based on the most recent Joint Interim Statement (JIS) of the International Diabetes Federation Task Force on Epidemiology and Prevention; National Heart, Lung, and Blood Institute; American Heart Association; World Heart Federation; International Atherosclerosis Society; and International Association for the Study of Obesity^16^ by adopting the Asian criteria for waist circumference. Participants were classified as having MS if they had at least 3 of following metabolic risk factors: central obesity (waist circumference ≥ 85 cm for Chinese men and ≥ 80 cm for Chinese women; elevated TG (fasting serum TG ≥ 1.7 mmol/L or taking medication for abnormal lipid levels); reduced HDL-c (fasting serum HDL-c < 1.0 mmol/L for Chinese men and < 1.3 mmol/L for Chinese women or specific treatment for this lipid abnormality); elevated blood pressure (SBP ≥ 130 mmHg or DBP ≥ 85 mmHg or taking hypertension medication); elevated fasting glucose (serum glucose level ≥ 5.6 mmol/L or taking diabetes medication).

### Statistical analysis

Continuous variables were reported as the mean ± standard deviation (SD) and compared using ANOVA test, and categorical variables were reported as percentages and compared using Pearson chi-square test, between non-MS cases and MS cases in both genders. Odds Ratios (ORs) and 95% confidence intervals (95% CIs) were estimated using conditional logistic regression with condition on two stages to assess the associations between MS with PAs, familial history of diseases, smoking status and alcohol consumption. The regression models were adjusted for the potential confounders, including age at interview (continuous), BMI (continuous), marital status, occupation, education level, and mutually adjusted for ever smoker, ever drinker, PA and risk scores of familial history of diseases in our calculation. The test for linear trend was performed by entering the ordinal exposure (such as median of each category in smoking (cigars per day), alcohol consumption and PA) as continuous parameters in the models. The SAS statistical software (version 9.4; SAS Institute, Cary, NC) was used for statistical analyses. All *p* values presented were based on two-tailed test, and *p* < 0.05 was considered statistically significant.

## Results

In current study, the overall prevalence of MS was 42.6% (45.0% in female and 39.8% in male) in 2013, which is much higher than 21.6% (23.7% in female and 17.5% in male) in 2007–2008 in rural Nantong, China. The study also showed that significantly higher prevalence of MS in older people among men and women in each stage in Table 1. The MS prevalence increased from 14.7% and 10.2% in age of less than 30 years to 51.5% and 61.5% in age of 60–69 years in both men and women in 2013, respectively. However, there were no statistically significant difference of the MS rates between 60–69 years age group and above 70 years age group in women (p = 0.412). Similarly results were found in several MS components (glucose, SBP and DBP). It had the highest glucose level in the age of 60–69 years, the highest SBP in the age above 70 years, and the highest DBP in the age of 50–59 years in both men and women in each stage. The highest waist and TG were found in the age of 40–49 years and 50–59 years in men and women in each stage, respectively. But HDL-c was found significantly increased by age in stage I, and this trend did not occur in stage II.

The selected characteristics between MS cases and non-MS cases in both genders were summarized in Table 2. In brief, the rates of ever smoker between MS cases and non-MS cases were comparable after adjusting for age at interview in both genders in each stage. MS cases were older and had higher BMI, and were also more likely to have diabetes and CHD disease than non-MS cases in both genders in each stage. Male MS cases were more educated and had a higher marital rate, and less likely to be farmers, compared with non-MS men in each stage. But in women MS cases were less educated and more likely to be farmers in stage II only. Additionally, MS cases had more exercise in both genders in stage II, Male MS cases were more likely to be alcohol consumers in stage II while female MS cases were less likely to be drinkers in stage I.

Table 3 presents associations of familial history of diseases (including hypertension, diabetes, hyperlipidemia, CHD and stroke in our study) and PAs (including LPA, OPA, hours of vigorous-intensity and moderate-intensity PA of both LPA and OPA) with MS adjusted for potential confounders. Familial history of diseases was associated with increased 20–90% of MS risk, compared with those people without these diseases in their familial history. Also, increasing risk scores, composed of number of these diseases of familial history, was associated with increased ORs of MS (p < 0.0001) in both genders. There were no association of LPA and the risk of having MS, however moderate to vigorous intensity OPA were associated with a decreased MS prevalence (ORs: 0.74 and 0.54 in men, and 0.69 and 0.73 in women, respectively). For the light intensity OPA, it may decrease 16% of MS risk in women only, compared with those without OPA or having sedentary work. Furthermore, increasing MET-h/w of total PA was significantly associated with a decreased risk of MS in both men and women, with ORs of 0.76 (95% CI: 0.71–0.81) for men and 0.81 (95% CI: 0.76–0.86) for women. Similar results were observed if we used hours/week (h/w) for vigorous-intensity or moderate-intensity PA in the analysis. Vigorous-intensity PA (>19 h/w in men and >28 h/w in women) was associated with 36 and 21% decreased MS risk, while moderate-intensity PA (>21 h/w in men and >17.5 h/w in women) was associated with equally decreased MS risk of 21% for men and women, compared with those people without vigorous- or moderate-intensity PA.

Table 4 presents the relationship of MS with cigarette smoking and alcohol consumption. There was no association of cigarette smoking with MS in men after adjustment for multiple confounders. However, we observed that in women current smokers were associated with risk of MS. Women who smoked 10 or more cigarettes per day were associated with 96% of increased MS risk (p = 0.0046 for trend), or who smoked over 25 years were associated with 74% of increased MS risk, compared with women non-smokers. In general, women drinkers, no matter what kind of alcoholic beverage they consumption, were associated with lower risk of having MS with OR of 0.70 (95% CI: 0.69–0.82). If we combine alcohol intake of beer, liquor and rice wine together, we observed that increasing a women’s alcohol consumption was associated with 15% decreased risk of MS (p < 0.0001 for the trend). Both number of month and amount of rice wine consumption were inversely associated with MS risk with OR of 0.72 (95% CI: 0.62–0.82) and 0.80 (0.73–0.88), respectively. A similar but weak association pattern was found in months and amount of beer consumption in women. Generally, we didn’t find any association of all source of alcohol consumption with MS in men. However, we do find a decreased risk of having MS among rice wine consumers with OR of 0.85 (95% CI: 0.77–0.95) for months of rice wine consumed and 0.91 (0.84–0.98) for amount of rice wine consumption in men. Also liquor was not associated with the prevalence of MS in both genders in our study.

**Figure 1:**
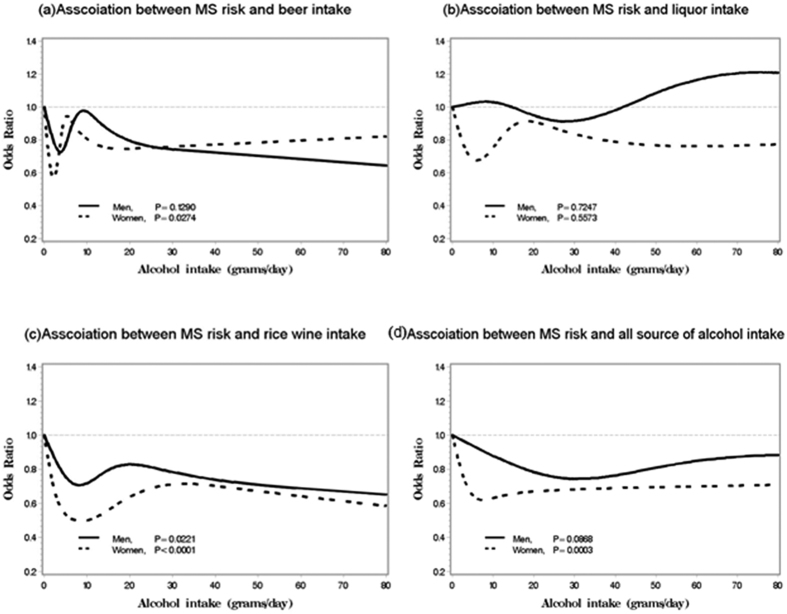
Association between alcohol consumption and MS risk in both men and women. Legend: associations between the MS risk and various alcoholic beverage intakes in 14,802 men and 22,780 women conducted in rural China, and all p values were from liner trend. (**a**) Beer intake; (**b**) liquor intake; (**c**) rice wine intake; (**d**) all source of alcohol intake.

Figure 1 depicted the associations between beer, liquor, rice wine and all alcoholic beverage with MS risk in both men and women. We found alcoholic intakes had protective effect on the risk of MS (p = 0.0003) only in women and this protective effect was strongest at about 10–15 grams alcohol consumed per day and then leveled off when alcohol intake was more than 15 grams per day. The similar protective effect was found in beer and rice wine consumption. In particular, we observed that protective effect of rice wine on MS was gradually increased in both men and women when alcohol consumption was less than 10 grams per day and this effect was stable after 10 grams of rice wine intake.

## Discussion

In our study overall prevalence of MS in 2013 was 42.6%, which was about 2 times higher than that in 2007. The prevalence was significantly higher in elder people in both genders, and was higher in elder women than in elder man (above 50 years) in both stage I and II. We found that current smokers in women and familial history of hypertension, diabetes, hyperlipidemia, CHD and stroke in both genders were associated with the prevalence of MS, while rice wine consumption and moderate to vigorous intensity OPA were inversely associated with MS risk.

The prevalence of MS in our study was higher than that in previous reports in China: it was 4.9% to 7.9% in East China in 2004 to 2005^21^, 7.3% in South China in 2002^8^, and 7.9% to 15.1% in Northwest China in 2010^2^. Furthermore we reported the MS prevalence in 2007–2008 was 21.6% in East China, which was in line with a report of 22.4% in Northeast China^22^ and in urban Shanghai, East China in 2008^23^. However, our data is lower than that in rural Handan of China in 2006–2007 in which the prevalence of MS was 39.7% and 54.2% in men and women respectively^24^. The MS prevalence was increased rapidly and it was 42.6% in East China in 2013, which is comparable with some developed countries or areas, such as rural USA (39.9%) and urban USA (32.8%)^25^. The high prevalence of MS in our study could be related with high prevalence of obesity, diabetes, dyslipidemia and hypertension due to changes of lifestyles and food intakes in China^26^,^27^. In consistent with most of these previous studies^2^,^4^,^25^, we found that the MS incidence was more common in female (45.0% and 23.7% in 2013 and 2007-2008, respectively) than in male (that is 39.8 and 17.5%) in rural areas of East China.

We found that familial history of diseases, including hypertension, hyperlipidemia (female only), diabetes, CHD or stroke, was independent risk factor of MS and increased about 20–80% risk of MS. Among these genetic predisposing factors that affect the prevalence of MS, Paek *et al*. found that familial histories of hypertension and stroke were risk factors for both men and women, but diabetes and cardiovascular disease were risk factor only for women^28^. Mattsson *et al*. reported that familial histories of hypertension and diabetes are two determinants of MS in a 21 years follow-up prospective cohort study^29^. The different findings of each study may be related with individual components of MS^30^, Pei *et al*. reported that apolipoprotein B is an important marker to segregate individual with MS in Chinese families with familial combined hyperlipidemia^31^. A meta-analysis found that fat mass and obesity related gene FTO plays a critical role in leading to MS^32^. Also, we found having two or more of those familial histories of diseases increased 82 and 69% risk of MS in men and women, respectively, compared with those people without any of these diseases of familial histories, which is in line with previous researches, in which they reported that having three or more of these diseases of familial histories or having familial history of dyslipidemia were both associated with MS components^33^. Underline mechanism could be that these different diseases of familial history may share genetic polymorphisms related to MS component level and/or that different genetic polymorphisms related to these different diseases of familial history influence MS component level. However, MS is a complex disorder, the mechanism of genetic variants and interaction between genetic variants and environmental factors still need for further studies.

Previous studies have revealed that PA was associated with lower prevalence of MS^6^,^17^. Besides, increasing MET of PA or longer durations of moderate and vigorous PA were associated with a reduced occurrence of MS^34^. We also found this association in the rural areas of East China. The role of LPA in MS is still inconclusive. One previous study identified that an inverse association between light LPA and MS in overweight adults, and light- or moderate-intensity LPA have higher risk of MS than vigorous-intensity LPA in normal-weight adults^35^. A meta-analysis with 10-years follow-up study reported that a 29 and 32% lower risk of MS in men and women associated with the highest levels of LPA, but a weaker association between MS and moderate LPA in men, compared with those in the lowest levels of LPA^36^. However, our study was consistent with both Silveira’s and Dalacorte’s studies^37^,^38^, in which they did not find any association between LPA and MS in both genders. In our study, we found protective effects of moderate- to vigorous-intensity OPA on MS in both genders and light-intensity OPA could lead to decrease MS risk only in women, compared with people with no or sedentary work. It was consistent with Chu and Mabry’s studies that OPA is associated with substantial reduction in the prevalence of MS^39^,^40^. However, in Halldin *et al*.’s study they didn’t find any associations between OPA and MS in Stockholm in Sweden^41^. A possible explanation of the association is that in rural areas in China, farmers always have a moderate- or high-intensive OPA, and their energy expenditure mainly derived from OPA, therefore association between MS and OPA could be found. In contrast, in our study only 18.0% of participants took LPA, most of whom took light intensity LPA, therefore association between MS and LPA is difficult to be found. A possible mechanism of OPA effect on MS could be that PA is known to protect against age-related vascular endothelial dysfunction, and long-term moderate PA may lower systemic oxidative stress. Also, nitric oxide appears to play a role in exercise-induced cardio benefits, such as lowering blood pressure through dilation of blood vessels^42^. In addition, since even non-vigorous activity is associated with higher insulin sensitivity, PA may thereby lower the risk of MS via a similar biological mechanism^43^.

Previous studies have shown conflicting results regarding the influence of smoking on the prevalence of MS. Some studies have reported ever and current smokers were contributed to a higher risk of MS^44^,^45^. While no association was found in rural Northwest China^2^ and in men in urban China^6^ or even it was a negatively association between smokers and MS risk in rural Northeast China^46^, compared with non-smokers. Most of these studies had small sample sizes and used only univariate analysis without adjustment for possible confounders. Our study had a much larger sample size and adjustment for potential confounders. We found that smoking was an independent risk factor for MS in women, and female smokers who smoked more than 10 cigarettes per day or over 25 years were associated with 96% or 74% increased MS risk, compared with non-smokers. Underline mechanics of this association may be related to obesity because a study reported that smoking could increase the risk of abdominal obesity^47^ and insulin resistance^48^.

The influence of alcohol consumption on MS is complicated. A meta-analysis of 6 prospective studies reported that beer or spirits alcohol consumption was associated with an increased risk of MS while wine was associated with a reduced risk of MS in a systematic review^49^ and in USA^50^. A cohort study in Spain reported that wine or liquor consumption was non-significant association with MS, while beer consumption was associated with higher risk for MS (p for trend = 0.027)^51^. In our study, we found that total alcohol beverage consumption was associated with decreasing risk of MS under a J-sharped relationship (p = 0.0003 for trend) only in women. Similar results were found in rice wine, i.e. its consumption associated with decreasing risk of MS in both genders: rice wine consumption of less than 90 grams per day was inversely associated with the prevalence of MS and this protective effect remained if rice wine consumption was more than 90 grams per day. Also, Beer consumption of more than 3 months had a 27 and 39% decreased risk of MS in men and women. No association was found in liquor consumption. The mechanism of the protective effect could be that the polyphenols enriched in red wine possess multiple benefits on MS through their anti-oxidant, anti-inflammatory, vascular-protective and insulin-sensitizing properties^52^. Resveratrol, a polyphenolic compound enriched in red wine, combating the ageing process induced by nutrient excess^53^. In addition, red wine have higher levels of bioflavonoids, which may induce endothelium-dependent dilation of blood vessels and suppress the synthesis of endothelin-1 (ET-1), a peptide that has a vasoconstricting effect^54^. Chinese rice wine, a widely consumed alcohol by residents in rural China, contains large amount of polyphenol and bioflavonoids substance, which may partially explain association of rice wine consumption and low risk of MS.

The strengths of this study include a large sample size in rural East China, and a comprehensive data collected to detect association of MS with PA and alcoholic beverage by different kinds, and extensive information on confounders. However, limitations should take into account. First, the cross-sectional study precludes any casual conclusion and further longitudinal studies should be conducted to confirm the causality relationship. Second, there are 5,586 participants, who had no information of familial history, were excluded from familial history analysis in our study. This exclusion could make a bias in OR estimation. Finally, some of younger males worked out of area during enrollment and cannot be included in this study. It could affect our results, especially in the analysis of younger people.

## Conclusions

This cross-sectional study shows that the prevalence of MS was increasing rapidly from 21.6% in 2007–2008 to 42.6% in 2013, which is both much higher in women than that in men in rural areas of East China. Smoking in women and familial history of hypertension, hyperlipidemia, diabetes, CHD and stroke aggravated the prevalence of MS. While moderate to vigorous intensity OPA and rice wine consumption in both genders were associated with decreasing the risk of MS. Our findings implicate that OPA and rice wine consumption may possibly contribute to the prevention of MS in rural areas in developing countries, and more preventive efforts should be focused on the individuals with familial history of disease and smokers in rural population.

## Additional Information

**How to cite this article**: Xiao, J. *et al*. Prevalence of metabolic syndrome and its risk factors among rural adults in Nantong, China. *Sci. Rep.* **6**, 38089; doi: 10.1038/srep38089 (2016).

**Publisher's note:** Springer Nature remains neutral with regard to jurisdictional claims in published maps and institutional affiliations.

## Figures and Tables

**Table 1:** Prevalence of MS and its components in different age groups by two stages ( 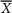 ± SD).

Age (years)	Stage I (6997 men and 13505 women)							Stage II (7805 men and 9275 women)						
	<30	30–39	40–49	50–59	60–69	≥70	p	<30	30–39	40–49	50–59	60–69	≥70	p
**Men**														
MS (n, %)	16 (5.3)	68 (13.8)	197 (18.9)	373 (17.9)	457 (19.4)	112 (15.4)	<0.001	84 (14.7)	162 (22.0)	578 (33.3)	731 (45.6)	881 (51.5)	669 (46.3)	<0.001
Waist (cm)	78.3 ± 10.7	83.2 ± 10.5	84.5 ± 10.2	83.7 ± 10.0	82.4 ± 10.4	80.7 ± 10.4	<0.001	83.1 ± 9.5	84.9 ± 9.1	85.7 ± 8.5	85.6 ± 8.5	85.4 ± 9.1	83.1 ± 9.3	<0.001
TG (mmol/L)	1.2 ± 1.1	1.7 ± 1.8	1.8 ± 2.0	1.5 ± 1.5	1.3 ± 1.1	1.1 ± 0.9	<0.001	1.4 ± 1.2	1.6 ± 1.3	1.6 ± 1.2	1.6 ± 1.2	1.6 ± 1.1	1.4 ± 0.9	<0.001
HDL-c (mmol/L)	1.4 ± 0.4	1.4 ± 0.4	1.5 ± 0.4	1.6 ± 0.4	1.6 ± 0.4	1.7 ± 0.4	<0.001	1.3 ± 0.4	1.3 ± 0.4	1.3 ± 0.4	1.3 ± 0.5	1.4 ± 0.5	1.4 ± 0.5	0.729
Glucose (mmol/L)	4.0 ± 0.5	4.2 ± 0.8	4.5 ± 1.1	4.5 ± 1.7	4.6 ± 2.1	4.4 ± 1.2	<0.001	5.0 ± 0.9	5.3 ± 1.3	5.4 ± 1.3	5.6 ± 1.5	5.7 ± 1.6	5.6 ± 1.4	<0.001
SBP (mmHg)	121.2 ± 12.0	121.4 ± 13.8	122.5 ± 14.8	124.6 ± 16.3	127.4 ± 19.2	129.0 ± 20.1	<0.001	121.3 ± 9.7	123.9 ± 10.9	128.0 ± 13.5	132.7 ± 15.7	137.3 ± 18.6	139.9 ± 20.4	<0.001
DBP (mmHg)	72.0 ± 9.1	74.6 ± 10.3	77.0 ± 11.2	77.0 ± 11.0	75.3 ± 10.8	73.6 ± 10.8	<0.001	75.4 ± 7.1	77.5 ± 7.6	79.7 ± 9.2	81.7 ± 10.1	80.8 ± 10.3	79.0 ± 10.7	<0.001
**Women**														
MS (n, %)	13 (2.7)	122 (8.7)	533 (17.4)	1176 (27.4)	1116 (32.0)	239 (30.5)	<0.001	73 (10.2)	186 (18.4)	798 (35.3)	946 (52.6)	1074 (61.5)	1095 (62.9)	<0.001
Waist (cm)	72.6 ± 8.2	76.1 ± 8.7	80.1 ± 9.7	82.7 ± 10.0	82.6 ± 10.7	81.2 ± 11.4	<0.001	77.1 ± 8.8	79.3 ± 8.5	82.5 ± 8.6	84.6 ± 9.2	84.2 ± 9.7	82.4 ± 9.8	<0.001
TG (mmol/L)	1.0 ± 0.7	1.2 ± 1.1	1.3 ± 1.2	1.6 ± 1.3	1.5 ± 1.2	1.4 ± 1.0	<0.001	1.2 ± 0.8	1.4 ± 0.9	1.5 ± 1.0	1.7 ± 1.3	1.7 ± 1.4	1.6 ± 1.1	<0.001
HDL-c (mmol/L)	1.5 ± 0.3	1.5 ± 0.3	1.5 ± 0.4	1.6 ± 0.4	1.6 ± 0.4	1.6 ± 0.4	<0.001	1.3 ± 0.3	1.3 ± 0.5	1.3 ± 0.4	1.4 ± 0.4	1.4 ± 0.6	1.4 ± 0.5	0.583
Glucose (mmol/L)	4.0 ± 0.5	4.2 ± 0.7	4.4 ± 1.3	4.6 ± 1.5	4.7 ± 1.6	4.7 ± 2.3	<0.001	5.0 ± 1.0	5.3 ± 1.3	5.5 ± 1.5	5.7 ± 1.7	5.8 ± 1.6	5.7 ± 1.5	<0.001
SBP (mmHg)	108.1 ± 9.8	111.0 ± 12.6	116.3 ± 15.8	122.3 ± 18.4	128.1 ± 20.1	131.3 ± 20.3	<0.001	116.8 ± 10.2	119.8 ± 12.6	125.5 ± 14.5	131.8 ± 17.1	136.8 ± 19.1	140.6 ± 20.9	<0.001
DBP (mmHg)	68.5 ± 7.8	70.6 ± 9.7	73.2 ± 10.6	74.4 ± 10.9	74.4 ± 10.9	73.2 ± 10.8	<0.001	73.4 ± 6.8	75.4 ± 7.8	77.7 ± 15.9	79.1 ± 9.9	78.7 ± 10.0	77.9 ± 10.4	<0.001

**Table 2:** Characteristics of MS cases and healthy individuals by two stages.

	Men (Stage I)			Men (Stage II)			Women (Stage I)			Women (Stage II)		
	Cases	Non-cases	p	Cases	Non-cases	p	Cases	Non-cases	p	Cases	Non-cases	p
	n = 1223	n = 5774		n = 3105	n = 4700		n = 3199	n = 10306		n = 4172	n = 5103	
Age (years, 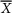 ± SD)	57.3 ± 10.6	55.8 ± 12.7	<0.001	58.6 ± 13.4	52.2 ± 16.5	<0.001	57.4 ± 9.4	51.9 ± 12.1	<0.001	59.9 ± 13.7	49.1 ± 16.4	<0.001
Height (cm, 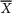 ± SD)^1^	167.2 ± 6.6	165.7 ± 6.6	<0.001	166.3 ± 7.0	165.4 ± 7.1	<0.001	156.3 ± 5.9	155.6 ± 6.1	<0.001	155.6 ± 7.1	155.6 ± 6.8	0.643
Weight (kg, 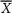 ± SD)^1^	74.8 ± 10.1	62.0 ± 10.0	<0.001	73.8 ± 10.8	65.1 ± 9.7	<0.001	65.5 ± 9.5	55.9 ± 9.0	<0.001	64.1 ± 10.3	58.1 ± 9.4	<0.001
BMI ( 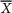 ± SD)^1^	26.7 ± 3.0	22.5 ± 3.1	<0.001	26.3 ± 3.3	23.8 ± 3.0	<0.001	26.8 ± 3.3	23.0 ± 3.3	<0.001	26.5 ± 3.7	24.0 ± 3.4	<0.001
Education (%)^1^												
Primary/under	40.8	54.2		45.2	49.2		64.6	63.7		65.4	60.7	
Middle	35.7	29.5		40.7	37.5		25.6	26.7		28.9	30.7	
High/above	23.5	16.3	<0.001	14.1	13.3	<0.001	9.8	9.6	0.969	5.7	8.6	<0.001
Marital status (%)^1^												
Yes	92.8	89.3		89.0	84.9		90.8	90.1		85.3	83.7	
No^2^	7.2	10.8	0.002	11.0	15.2	<0.001	9.2	9.9	0.808	14.7	16.3	0.114
Farmer (%)^1^												
Yes	46.1	59.3		62.5	63.6		69.4	69.6		77.3	73.8	
No	53.9	40.7	<0.001	37.5	36.4	0.069	30.7	30.4	0.413	22.7	26.2	0.002
Ever smoker (%)^1^												
Yes	45.8	48.1		49.7	48.5		3.6	4.0		2.2	1.9	
No	54.1	51.9	0.125	50.3	51.5	0.348	96.4	96.0	0.168	97.8	98.1	0.399
Ever drinker (%)^1^												
Yes	52.6	53.9		52.4	46.6		9.2	12.0		6.4	6.2	
No	47.4	46.1	0.223	47.6	53.4	<0.001	90.8	88.0	<0.001	93.6	93.8	0.741
Exercise (%)^1^												
Yes	22.5	21.7		18.4	13.1		20.4	20.4		14.7	12.7	
No	77.5	78.3	0.615	81.6	86.9	<0.001	79.6	79.6	0.533	85.3	87.3	0.009
Diabetes (%)^1^												
Yes	9.7	0.9		8.6	1.0		7.4	0.6		9.8	1.0	
No	90.3	99.1	<0.001	91.4	99.0	<0.001	92.6	99.4	<0.001	90.2	99.0	<0.001
CVD (%)^1^												
Yes	2.8	1.1		7.6	3.2		0.9	0.4		6.4	3.6	
No	97.2	98.9	<0.001	92.4	96.8	<0.001	99.1	99.6	0.002	93.6	96.4	<0.001

^1^means, percentages, and their p values were adjusted for age.

^2^Including widowed, divorced/separated and unmarried.

**Table 3:** Association of familial history of diseases and physical activities with prevalence of MS.

	Men					Women		
	Percentages of cases (%)		OR (95% CI)^a^	OR (95% CI)^b^		Percentages of cases (%)	OR (95% CI)^a^	OR (95% CI)^b^
**Familial history**								
Hypertension								
No	26.50		1.0	1.0		29.88	1.0	1.0
Yes	38.08		1.67 (1.51−1.85)	1.58 (1.43−1.75)		38.46	1.57 (1.44−1.70)	1.57 (1.44−1.70)
Hyperlipidemia								
No	17.90		1.0	1.0		22.86	1.0	1.0
Yes	31.71		1.33 (0.61−2.90)	1.21 (0.55−2.65)		33.33	1.90 (1.17−3.08)	1.88 (1.15−3.06)
Diabeles								
No	28.82		1.0	1.0		31.37	1.0	1.0
Yes	43.22		1.53 (1.21−1.93)	1.46 (1.15−1.84)		43.90	1.79 (1.48−2.18)	1.78 (1.46−2.16)
CHD								
No	28.85		1.0	1.0		31.51	1.0	1.0
Yes	36.89		1.35 (1.12−1.62)	1.31 (1.08−1.57)		37.36	1.18 (1.00−1.39)	1.18 (1.00−1.40)
Stroke								
No	28.13		1.0	1.0		30.94	1.0	1.0
Yes	45.13		1.51 (1.28−1.77)	1.46 (1.24−1.71)		46.77	1.51 (1.30−1.75)	1.51 (1.30−1.75)
Risk score								
0	25.64		1.0	1.0		29.22	1.0	1.0
1	34.87		1.55 (1.40−1.72)	1.49 (1.34−1.65)		36.76	1.53 (1.41−1.67)	1.53 (1.40−1.66)
≥2	45.80		1.94 (1.66−2.28)	1.82 (1.55−2.14)		44.48	1.69 (1.47−1.94)	1.69 (1.47−1.94)
p for trend			<0.0001	<0.0001			<0.0001	<0.0001
**Physical activity**								
LPA								
No		28.24	1.0	1.0		32.16	1.0	1.0
Yes		28.36	0.75 (0.66−0.85)	0.88 (0.75−1.04)		27.80	0.87 (0.79−0.96)	0.90 (0.80−1.01)
LPA (categories)								
No		28.24	1.0	1.0		32.16	1.0	1.0
Light-intensity		32.02	1.47 (1.27−1.69)	1.21 (1.00−1.46)		30.29	1.19 (1.06−1.35)	1.15 (0.99−1.34)
Moderate-intensity		20.32	1.08 (0.85−1.37)	1.03 (0.76−1.39)		23.87	1.06 (0.91−1.24)	1.05 (0.88−1.26)
Vigorous-intensity		20.78	1.02 (0.54−1.92)	0.86 (0.44−1.71)		30.14	1.30 (0.87−1.95)	1.27 (0.82−1.95)
*p* for trend			0.0011	0.3900			0.0231	0.1174
OPA								
No or sedentary work		33.76	1.0	1.0		34.06	1.0	1.0
Light-intensity		33.22	0.94 (0.82−1.08)	0.91 (0.79−1.05)		23.70	0.85 (0.72−1.00)	0.84 (0.71−1.00)
Moderate-intensity		15.15	0.71 (0.57−0.89)	0.74 (0.59−0.93)		13.90	0.72 (0.59−0.88)	0.69 (0.56−0.85)
Vigorous−intensity		14.71	0.49 (0.42−0.57)	0.54 (0.46−0.64)		25.82	0.71 (0.63−0.80)	0.73 (0.64−0.84)
*p* for trend			<0.0001	<0.0001			<0.0001	<0.0001
Vigorous-intensity PA (h/w)								
0		34.95	1.0	1.0	0	36.48	1.0	1.0
≤19		29.08	0.91 (0.79−1.05)	0.93 (0.81−1.07)	≤28	28.68	0.90 (0.81−0.99)	0.96 (0.86−1.07)
>19		16.87	0.63 (0.56−0.71)	0.64 (0.56−0.72)	>28	23.88	0.81 (0.73−0.90)	0.79 (0.70−0.89)
*p* for trend			<0.0001	<0.0001			0.0002	0.0001
Moderate-intensity PA (h/w)								
0		27.28	1.0	1.0	0	30.57	1.0	1.0
≤21		32.66	1.06 (0.96−1.18)	1.05 (0.95−1.17)	≤17.5	36.98	1.09 (1.00−1.19)	1.10 (1.00−1.21)
>21		25.10	0.86 (0.77−0.95)	0.79 (0.70−0.88)	>17.5	28.43	0.83 (0.76−0.90)	0.79 (0.73−0.87)
*p* for trend			0.0102	0.0001			<0.0001	<0.0001
Total PA (MET-h/w)								
≤110.9		40.61	1.0	1.0	≤130.0	45.11	1.0	1.0
≤245.3		28.19	0.81 (0.73−0.90)	0.79 (0.70−0.88)	≤268.8	25.42	0.71 (0.65−0.80)	0.71 (0.64−0.79)
>245.3		15.81	0.55 (0.48−0.63)	0.57 (0.49−0.66)	>268.8	23.89	0.63 (0.56−0.71)	0.64 (0.56−0.72)
*p* for trend			<0.0001	<0.0001			<0.0001	<0.0001

^a^Adjusted for age at interview and BMI.

^b^Adjusted for age at interview, BMI, education level, marital status, ever smoker and ever drinker, additionally

adjusted for occupation and exercise in familial history calculation and adjusted for risk scores of familial history in physical activity calculation, and mutually adjusted for moderate and vigorous PA or LPA and OPA categories. LPA: Leisure-time physical activity. OPA: Occupational physical activity. *p* for trend was calculated by entering the ordinal exposure as continuous parameters in the model.

**Table 4:** Association of smoking and alcohol consumption with prevalence of MS.

	Men				Women		
	Percentages of Case (%)	OR (95% CI)^a^	OR (95% CI)^b^		Percentages of Case (%)	OR (95% CI)^a^	OR (95% CI)^b^
Smoking status							
Never	21.89	1.0	1.0		25.72	1.0	1.0
Former	19.23	1.00 (0.88−1.13)	1.14 (0.97−1.35)		28.80	1.06 (0.85−1.33)	1.32 (1.00−1.73)
Current	25.48	1.16 (0.89−1.52)	1.24 (0.89−1.72)		32.84	1.49 (0.83−2.67)	2.39 (1.22−4.67)
*p* for trend		0.5284	0.0669			0.2307	0.0022
Smoking rate (cigars per day)							
Never	21.89	1.0	1.0	Never	25.71	1.0	1.0
≤10	17.04	0.87 (0.74−1.03)	1.01 (0.82−1.24)	≤5	27.84	1.02 (0.71−1.46)	1.21 (0.79−1.84)
≤20	21.37	1.12 (0.96−1.32)	1.15 (0.94−1.41)	≤10	29.15	1.06 (0.75−1.49)	1.34 (0.91−2.08)
>20	24.73	1.04 (0.70−1.55)	0.98 (0.60−1.62)	>10	35.29	1.53 (0.95−2.46)	1.96 (1.12−3.43)
*p* for trend		0.3261	0.1535			0.1576	0.0046
Years of smoke							
Never	21.91	1.0	1.0		25.72	1.0	1.0
≤25	17.35	0.90 (0.73−1.11)	1.01 (0.79−1.30)		24.69	0.81 (0.52−1.21)	0.85 (0.53−1.40)
>25	20.05	1.01 (0.88−1.17)	1.11 (0.93−1.33)		31.53	1.26 (0.96−1.64)	1.74 (1.26−2.40)
*p* for trend		0.8818	0.2086			0.2067	0.0028
Ever drink							
Never	21.61	1.0	1.0		26.28	1.0	1.0
Ever	20.39	0.91 (0.81−1.03)	0.89 (0.76−1.04)		22.17	0.68 (0.59−0.78)	0.70 (0.69−0.82)
Beer: months of drink							
0	22.75	1.0	1.0		26.15	1.0	1.0
≤3	17.04	0.75 (0.64−0.87)	0.94 (0.79−1.12)		22.76	0.77 (0.63−0.93)	0.87 (0.70−1.07)
>3	17.72	0.68 (0.55−0.85)	0.73 (0.56−0.94)		19.79	0.57 (0.41−0.79)	0.61 (0.42−0.88)
*p* for trend		<0.0001	0.0228			<0.0001	0.0050
Alcohol from beer (g/d)							
0	22.76	1.0	1.0	0	26.15	1.0	1.0
≤6.2	16.47	0.67 (0.57−0.80)	0.83 (0.68−1.01)	≤2.8	21.14	0.61 (0.46−0.82)	0.70 (0.51−0.97)
≤12.3	19.57	0.87 (0.62−1.23)	1.09 (0.74−1.59)	≤6.2	22.75	0.78 (0.62−0.99)	0.88 (0.68−1.13)
>12.3	17.57	0.77 (0.63−0.95)	0.88 (0.70−1.12)	>6.2	20.98	0.70 (0.48−1.02)	0.74 (0.49−1.13)
*p* for trend		0.0008	0.2737			0.0005	0.0312
Liquor: months of drink							
0	20.03	1.0	1.0		25.84	1.0	1.0
≤6	18.00	0.88 (0.73−1.05)	0.92 (0.74−1.14)		25.83	0.85 (0.64−1.13)	0.94 (0.68−1.30)
>6	29.98	1.44 (1.23−1.70)	1.11 (0.87−1.42)		27.53	0.91 (0.62−1.31)	0.76 (0.47−1.23)
*p* for trend		0.0009	0.6774			0.2973	0.2574
Alcohol from liquor (g/d)							
0	20.13	1.0	1.0	0	25.86	1.0	1.0
≤12.3	19.87	0.89 (0.60−1.13)	0.85 (0.64−1.14)	≤6.2	26.46	0.88 (0.61−1.27)	1.01 (0.67−1.54)
≤37	22.30	1.09 (0.89−1.32)	1.02 (0.80−1.30)	≤24.6	22.50	0.64 (0.40−1.02)	0.67 (0.40−1.14)
>37	27.68	1.35 (1.10−1.65)	1.08 (0.81−1.43)	>24.6	26.99	0.92 (0.62−1.36)	0.86 (0.53−1.40)
*p* for trend		0.0099	0.7096			0.1457	0.2280
Rice wine: months of drink							
0	23.26	1.0	1.0		26.27	1.0	1.0
≤6	16.46	0.68 (0.58−0.81)	0.85 (0.70−1.04)		21.40	0.60 (0.48−0.75)	0.68 (0.53−0.86)
>6	14.34	0.59 (0.48−0.73)	0.73 (0.58−0.93)		17.72	0.55 (0.42−0.74)	0.54 (0.39−0.75)
*p* for trend		<0.0001	0.0048			<0.0001	<0.0001
Alcohol from rice wine (g/d)							
0	23.26	1.0	1.0	0	26.27	1.0	1.0
≤8.6	15.74	0.61 (0.49−0.76)	0.80 (0.63−1.03)	≤4.1	21.54	0.61 (0.46−0.83)	0.77 (0.56−1.05)
≤25.7	15.34	0.67 (0.55−0.82)	0.83 (0.67−1.04)	≤12.8	18.65	0.52 (0.38−0.71)	0.49 (0.35−0.70)
>25.7	15.81	0.65 (0.51−0.84)	0.76 (0.57−1.00)	>12.8	19.35	0.61 (0.45−0.81)	0.63 (0.45−0.87)
*p* for trend		<0.0001	0.0152			<0.0001	<0.0001
All source of alcohol (g/d)							
0	21.91	1.0	1.0	0	26.31	1.0	1.0
≤16.4	20.23	0.89 (0.74−1.05)	0.97 (0.79−1.20)	≤5.6	22.31	0.66 (0.52−0.84)	0.73 (0.56−0.96)
≤45.2	18.43	0.79 (0.67−0.94)	0.78 (0.63−0.97)	≤18.5	21.06	0.64 (0.50−0.81)	0.67 (0.52−0.87)
>45.2	21.54	0.97 (0.82−1.15)	0.92 (0.73−1.16)	>18.5	22.33	0.69 (0.55−0.88)	0.68 (0.51−0.89)
*p* for trend		0.1619	0.1194			<0.0001	<0.0001

^a^Adjusted for age at interview and BMI.

^b^Adjusted for age at interview, BMI, education, marital status, occupation, exercise, risk scores of familial history, and mutually adjusted for ever smoker and ever drinker; *p* for trend was calculated by entering the ordinal exposure as continuous parameters in the model.
